# The genome sequence of the Bluebell moth, *Eana incanana* (Stephens, 1852) (Lepidoptera: Tortricidae)

**DOI:** 10.12688/wellcomeopenres.26549.1

**Published:** 2026-05-11

**Authors:** David C. Lees, Rachel Foster, Natural History Museum Genome Acquisition Lab, Darwin Tree of Life Barcoding Collective, Wellcome Sanger Institute Tree of Life Management, Samples and Laboratory team, Wellcome Sanger Institute Scientific Operations: Sequencing Operations, Wellcome Sanger Institute Tree of Life Core Informatics team, Tree of Life Core Informatics collective, Darwin Tree of Life Consortium

**Affiliations:** 1 Natural History Museum, London, England, UK

**Keywords:** Eana incanana, Bluebell moth, genome sequence, chromosomal, Lepidoptera

## Abstract

We present a genome assembly from an individual male *Eana incanana* (Bluebell moth; Arthropoda; Insecta; Lepidoptera; Tortricidae). The assembly contains two haplotypes with total lengths of 372.33 megabases and 373.40 megabases. Most of haplotype 1 (99.64%) is scaffolded into 30 chromosomal pseudomolecules, including the Z sex chromosome. Haplotype 2 was assembled to scaffold level. The mitochondrial genome has also been assembled, with a length of 16.01 kilobases. This assembly was generated as part of the Darwin Tree of Life project, which produces genomes for eukaryotic species found in Britain and Ireland.

## Species taxonomy

Eukaryota; Opisthokonta; Metazoa; Eumetazoa; Bilateria; Protostomia; Ecdysozoa; Panarthropoda; Arthropoda; Mandibulata; Pancrustacea; Altocrustacea; Allotriocarida; Hexapoda; Insecta; Dicondylia; Pterygota; Neoptera; Eumetabola; Endopterygota; Aparaglossata; Panorpida; Amphiesmenoptera; Lepidoptera; Glossata; Neolepidoptera; Heteroneura; Ditrysia; Apoditrysia; Tortricoidea; Tortricidae; Tortricinae; Cnephasiini; *Eana*; *Eana incanana* (Stephens, 1852) (NCBI:txid1594282).

## Background

The Bluebell Moth, *Eana incanana* (Stephens, 1852), is a micro-moth belonging to the family Tortricidae. The adult moth has an approximate length of 11.5 mm and a wingspan of 17–23 mm. The forewing is morphologically similar to larger species of *Cnephasia* Curtis, 1826, with predominant colours of silvery-grey, marbled with darker browns and sometimes smaller orange markings, it differs in the position and shape of the crossband from *C. stephensiana* (Doubleday, [1849]); examination of genitalia may be required to confirm its identity ( Sterling *et al.*, 2023).

*Eana incanana* is commonly found across northern Europe, with records also scattered as far east as Russia and Siberia ( GBIF Secretariat, 2023). It has also been recorded from Iran ( Alipanah, 2019) and Japan ( Nasu & Kogi, 2008).

The Bluebell moth is a single-brooded species that flies during June, July and August and comes to light. It is found in woodland habitats and pupates in leaf litter. The larvae of *Eana incanana* feed on flowers of the English Bluebell ( *Hyacinthoides non-scripta* (L.) Chouard ex Rothm.) and the Ox-eye Daisy ( *Leucanthemum vulgare* Lam.) ( Sterling *et al.*, 2023). The English Bluebell is known to be toxic to livestock due to the presence of defensive glycosidase inhibitors; however, *E. incanana* larvae can selectively metabolise a key glycosidase inhibitor (DMDP) while excreting others, uniquely allowing them to tolerate the plant’s chemical defences ( Watson *et al.*, 2008).

The DNA barcode from the mitogenome assembly presented here (OZ211728.1) belongs to BIN cluster BOLD:AAD7476. Within Europe, sequences in this cluster differ by no more than two nucleotides, and the nearest neighbouring BIN is *Eana penziana* (BOLD:AAB8228), with a minimum *p*-distance of 3.54%.

## Methods

### Sample acquisition and DNA barcoding

**Figure 1.**
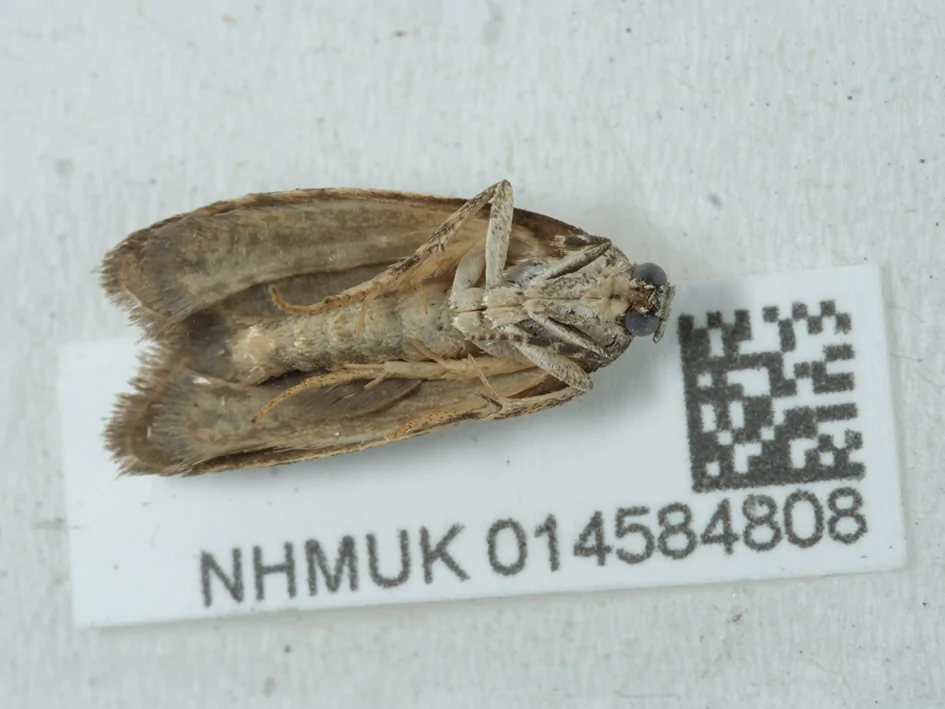
Photograph of the *Eana incanana* (ilEanInca1) specimen used for genome sequencing.

The specimen used for genome sequencing was an adult male *Eana incanana* (specimen ID NHMUK014584808, ToLID ilEanInca1; Figure 1), collected from the NHM Wildlife Garden, London, UK (latitude 51.5, longitude −0.18) on 2022-06-14. The specimen was collected and identified by David Lees (Natural History Museum London).

The initial identification was verified by an additional DNA barcoding process according to the framework developed by Twyford *et al.* (2024). A small sample was dissected from the specimen and stored in ethanol, while the remaining parts were shipped on dry ice to the Wellcome Sanger Institute (WSI) (see the protocol). The tissue was lysed, the COI marker region was amplified by PCR, and amplicons were sequenced and compared to the BOLD database, confirming the species identification ( Crowley *et al.*, 2023). Following whole genome sequence generation, the relevant DNA barcode region was also used alongside the initial barcoding data for sample tracking at the WSI ( Twyford *et al.*, 2024). The standard operating procedures for Darwin Tree of Life barcoding are available on protocols.io.

### Nucleic acid extraction

Detailed protocols for nucleic acid extraction developed at the Wellcome Sanger Institute (WSI) Tree of Life Core Laboratory are available on protocols.io ( Howard *et al.*, 2025). The ilEanInca1 sample was weighed and triaged to determine the appropriate extraction protocol. For HMW DNA extraction, 7 mg of tissue from the whole organism was used. Tissue from the whole organism was homogenised by powermashing using a PowerMasher II tissue disruptor. High molecular weight (HMW) DNA was extracted in the WSI Scientific Operations core using the Automated MagAttract v2 protocol. DNA was sheared into an average fragment size of 12–20 kb following the Megaruptor®3 for LI PacBio protocol. Sheared DNA was purified by automated SPRI (solid-phase reversible immobilisation). The concentration of the sheared and purified DNA was assessed using a Nanodrop spectrophotometer and Qubit Fluorometer using the Qubit dsDNA High Sensitivity Assay kit. Fragment size distribution was evaluated by running the sample on the FemtoPulse system. For this sample, the final post-shearing DNA had a Qubit concentration of 22.56 ng/μL and a yield of 1 060.32 ng, with a fragment size of 14.8 kb. The Genomic Quality Number (GQN) was 7.4.

### PacBio HiFi library preparation and sequencing

Library preparation and sequencing were performed at the WSI Scientific Operations core. Libraries were prepared using the SMRTbell Prep Kit 3.0 (Pacific Biosciences, California, USA), following the manufacturer’s instructions. The kit includes reagents for end repair/A-tailing, adapter ligation, post-ligation SMRTbell bead clean-up, and nuclease treatment. Size selection and clean-up were performed using diluted AMPure PB beads (Pacific Biosciences). DNA concentration was quantified using a Qubit Fluorometer v4.0 (ThermoFisher Scientific) and the Qubit 1X dsDNA HS assay kit. Final library fragment size was assessed with the Agilent Femto Pulse Automated Pulsed Field CE Instrument (Agilent Technologies) using the gDNA 55 kb BAC analysis kit.

The sample was sequenced on a Revio instrument (Pacific Biosciences). The prepared library was normalised to 2 nM, and 15 μL was used for making complexes. Primers were annealed and polymerases bound to generate circularised complexes, following the manufacturer’s instructions. Complexes were purified using 1.2X SMRTbell beads, then diluted to the Revio loading concentration (200–300 pM) and spiked with a Revio sequencing internal control. The sample was sequenced on a Revio 25 M SMRT cell. The SMRT Link software (Pacific Biosciences), a web-based workflow manager, was used to configure and monitor the run and to carry out primary and secondary data analysis.

### Hi-C

***Sample preparation and crosslinking***

The Hi-C sample was prepared from 20–50 mg of frozen whole organism tissue of the ilEanInca1 sample using the Arima-HiC v2 kit (Arima Genomics). Following the manufacturer’s instructions, tissue was fixed and DNA crosslinked using TC buffer to a final formaldehyde concentration of 2%. The tissue was homogenised using the Diagnocine Power Masher-II. Crosslinked DNA was digested with a restriction enzyme master mix, biotinylated, and ligated. Clean-up was performed with SPRISelect beads before library preparation. DNA concentration was measured with the Qubit Fluorometer (Thermo Fisher Scientific) and Qubit HS Assay Kit. The biotinylation percentage was estimated using the Arima-HiC v2 QC beads.

***Hi-C library preparation and sequencing***

Biotinylated DNA constructs were fragmented using a Covaris E220 sonicator and size selected to 400–600 bp using SPRISelect beads. DNA was enriched with Arima-HiC v2 kit Enrichment beads. End repair, A-tailing, and adapter ligation were carried out with the NEBNext Ultra II DNA Library Prep Kit (New England Biolabs), following a modified protocol where library preparation occurs while DNA remains bound to the Enrichment beads. Library amplification was performed using KAPA HiFi HotStart mix and a custom Unique Dual Index (UDI) barcode set (Integrated DNA Technologies). Depending on sample concentration and biotinylation percentage determined at the crosslinking stage, libraries were amplified with 10–16 PCR cycles. Post-PCR clean-up was performed with SPRISelect beads. Libraries were quantified using the AccuClear Ultra High Sensitivity dsDNA Standards Assay Kit (Biotium) and a FLUOstar Omega plate reader (BMG Labtech).

Prior to sequencing, libraries were normalised to 10 ng/μL. Normalised libraries were quantified again to create equimolar and/or weighted 2.8 nM pools. Pool concentrations were checked using the Agilent 4200 TapeStation (Agilent) with High Sensitivity D500 reagents before sequencing. Sequencing was performed using paired-end 150 bp reads on the Illumina NovaSeq X.

### Genome assembly

Prior to assembly of the PacBio HiFi reads, a database of *k*-mer counts ( *k* = 31) was generated from the filtered reads using FastK. GenomeScope2 ( Ranallo-Benavidez *et al.*, 2020) was used to analyse the *k*-mer frequency distributions, providing estimates of genome size, heterozygosity, and repeat content.

The HiFi reads were assembled using Hifiasm in Hi-C phasing mode ( Cheng *et al.*, 2021, 2022), producing two haplotypes. Hi-C reads ( Rao *et al.*, 2014) were mapped to the primary contigs using bwa-mem2 ( Vasimuddin *et al.*, 2019). Contigs were further scaffolded with Hi-C data in YaHS ( Zhou *et al.*, 2023), using the --break option for handling potential misassemblies. The scaffolded assemblies were evaluated using Gfastats ( Formenti *et al.*, 2022), BUSCO ( Manni *et al.*, 2021) and MerquryFK ( Rhie *et al.*, 2020).

The mitochondrial genome was assembled using MitoHiFi ( Uliano-Silva *et al.*, 2023).

### Assembly curation

The assembly was decontaminated using the Assembly Screen for Cobionts and Contaminants ( ASCC) pipeline. TreeVal was used to generate the flat files and maps for use in curation. Manual curation was conducted primarily in PretextView and HiGlass ( Kerpedjiev *et al.*, 2018). Scaffolds were visually inspected and corrected as described by Howe *et al*. (2021). Manual corrections included 14 breaks, 21 joins, and removal of 11 haplotypic duplications. This reduced the scaffold count by 24.3%. The curation process is described at https://gitlab.com/wtsi-grit/rapid-curation . PretextSnapshot was used to generate a Hi-C contact map of the final assembly.

### Assembly quality assessment

The MerquryFK tool ( Rhie *et al.*, 2020) was run in a Singularity container ( Kurtzer *et al.*, 2017) to evaluate *k*-mer completeness and assembly quality for both haplotypes using the *k*-mer database ( *k* = 31) computed prior to genome assembly. The analysis outputs included assembly QV scores and completeness statistics.

The genome was analysed using the BlobToolKit pipeline, a Nextflow implementation of the earlier Snakemake version ( Challis *et al.*, 2020). The pipeline aligns PacBio reads using minimap2 ( Li, 2018) and SAMtools ( Danecek *et al.*, 2021) to generate coverage tracks. It runs BUSCO ( Manni *et al.*, 2021) using lineages identified from the NCBI Taxonomy ( Schoch *et al.*, 2020). For the three domain-level lineages, BUSCO genes are aligned to the UniProt Reference Proteomes database ( Bateman *et al.*, 2023) using DIAMOND blastp ( Buchfink *et al.*, 2021). The genome is divided into chunks based on the density of BUSCO genes from the closest taxonomic lineage, and each chunk is aligned to the UniProt Reference Proteomes database with DIAMOND blastx. Sequences without hits are chunked using seqtk and aligned to the NT database with blastn ( Altschul *et al.*, 1990). The BlobToolKit suite consolidates all outputs into a blobdir for visualisation. The BlobToolKit pipeline was developed using nf-core tooling ( Ewels *et al.*, 2020) and MultiQC ( Ewels *et al.*, 2016), with containerisation through Docker ( Merkel, 2014) and Singularity ( Kurtzer *et al.*, 2017).

## Genome sequence report

### Sequence data

**Figure 2.**
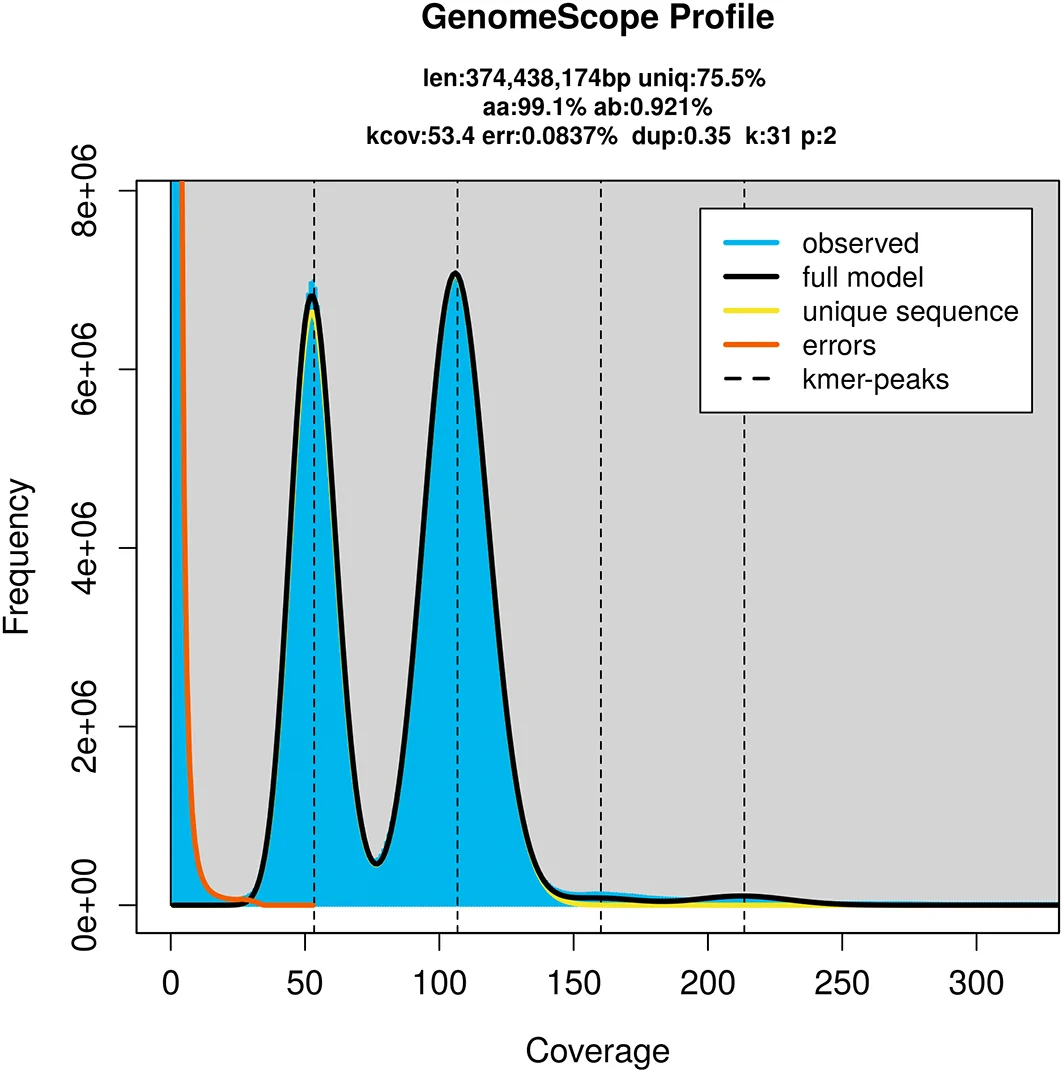
Frequency distribution of *k*-mers generated using GenomeScope2. The plot shows observed and modelled *k*-mer spectra, providing estimates of genome size, heterozygosity, and repeat content based on unassembled sequencing reads.

PacBio sequencing of the *Eana incanana* specimen generated 41.14 Gb (gigabases) from 3.54 million reads, which were used to assemble the genome. GenomeScope2.0 analysis estimated the haploid genome size at 374.44 Mb, with a heterozygosity of 0.92% and repeat content of 24.50% ( Figure 2). These estimates guided expectations for the assembly. Based on the estimated genome size, the sequencing data provided approximately 107× coverage. Hi-C sequencing produced 121.44 Gb from 402.13 million reads, which were used to scaffold the assembly. Table 1 summarises the specimen and sequencing details.

**Table 1.** Specimen and sequencing data for *Eana incanana* (bioProject PRJEB82141).

Platform	PacBio HiFi	Hi-C
**ToLID**	ilEanInca1	ilEanInca1
**Specimen ID**	NHMUK014584808	NHMUK014584808
**BioSample (source individual)**	SAMEA115575021	SAMEA115575021
**BioSample (tissue)**	SAMEA115575177	SAMEA115575177
**Tissue**	whole organism	whole organism
**Instrument**	Revio	Illumina NovaSeq X
**Run accessions**	ERR13946448	ERR13947467
**Read count total**	3.54 million	402.13 million
**Base count total**	41.14 Gb	121.44 Gb

### Assembly statistics

The genome was assembled into two haplotypes using Hi-C phasing. Haplotype 1 was curated to chromosome level, while haplotype 2 was assembled to scaffold level. The final assembly has a total length of 372.33 Mb in 52 scaffolds, with 70 gaps, and a scaffold N50 of 12.57 Mb ( Table 2).

**Table 2.** Genome assembly statistics for *Eana incanana.*

Genome assembly	Haplotype 1	Haplotype 2
**Assembly name**	ilEanInca1.hap1.1	ilEanInca1.hap2.1
**Assembly accession**	GCA_964662145.1	GCA_964662215.1
**Assembly level**	chromosome	scaffold
**Span (Mb)**	372.33	373.40
**Number of chromosomes**	30	scaffold-level
**Number of contigs**	122	118
**Contig N50**	6.34 Mb	7.93 Mb
**Number of scaffolds**	52	72
**Scaffold N50**	12.57 Mb	12.59 Mb
**Longest scaffold length (Mb)**	41.51	-
**Sex chromosomes**	Z	-
**Organelles**	Mitochondrion: 16.01 kb	-

**Figure 3.**
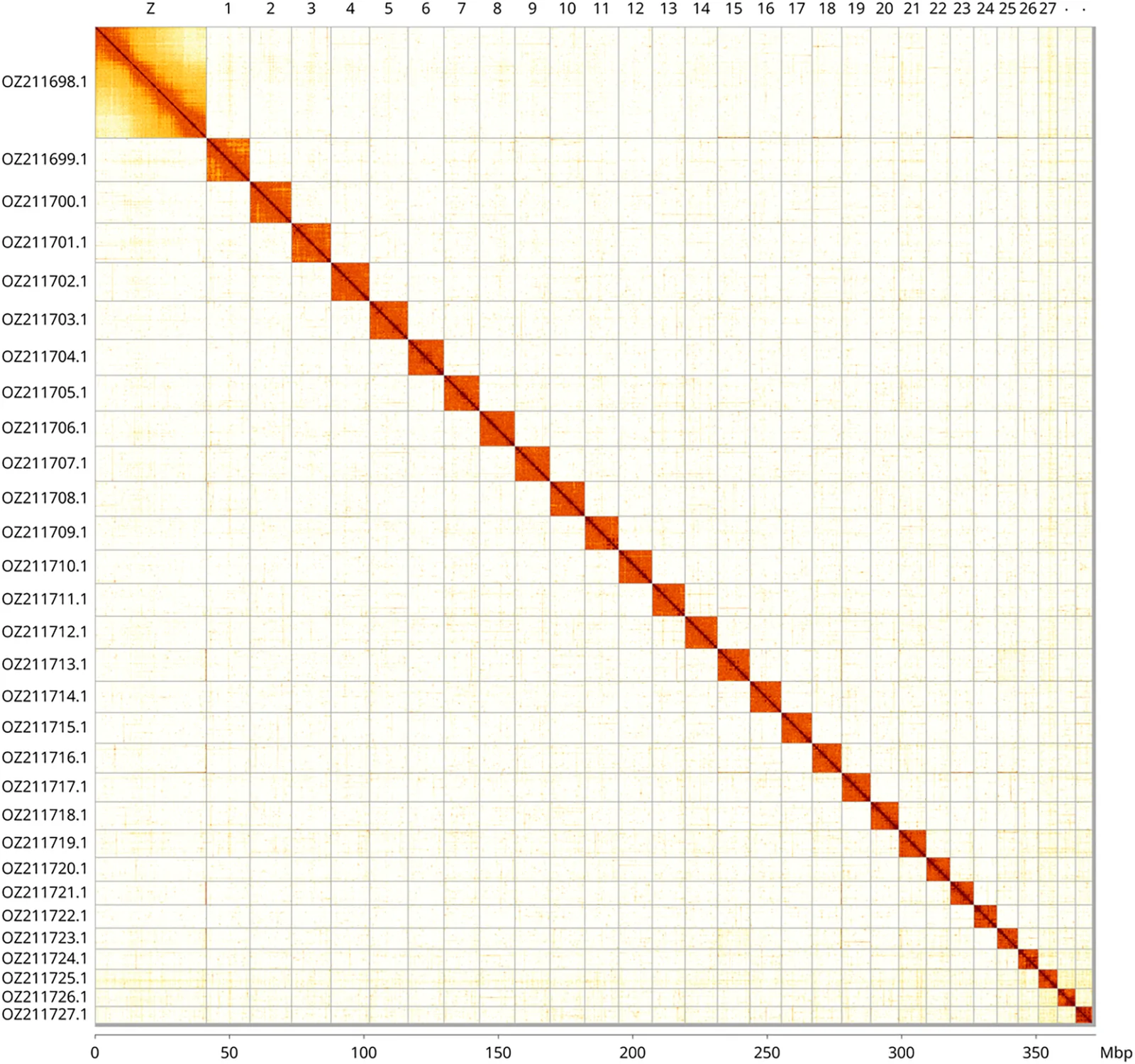
Hi-C contact map of the *Eana incanana* genome assembly. Assembled chromosomes are shown in order of size and labelled along the axes, with a megabase scale shown below. The plot was generated using PretextSnapshot.

Most of the haplotype 1 assembly sequence (99.64%) was assigned to 30 chromosomal-level scaffolds, representing 29 autosomes and the Z sex chromosome. These chromosome-level scaffolds, confirmed by Hi-C data, are named according to size ( Figure 3; Table 3). The Z chromosome was identified based on BUSCO gene painting with ancestral Merian elements ( Wright *et al.*, 2024). During curation, we observed that there is a haplotypic inversion on Chromosome Z in the region ~12.94–14.25 Mb.

**Table 3.** Chromosomal pseudomolecules in the haplotype 1 genome assembly of *Eana incanana* ilEanInca1.

INSDC accession	Molecule	Length (Mb)	GC%
OZ211699.1	1	16.15	37.50
OZ211700.1	2	15.52	37
OZ211701.1	3	14.66	37.50
OZ211702.1	4	14.36	36.50
OZ211703.1	5	14.25	37
OZ211704.1	6	13.40	36.50
OZ211705.1	7	13.23	36.50
OZ211706.1	8	13.15	37
OZ211707.1	9	13.05	37
OZ211708.1	10	12.96	37.50
OZ211709.1	11	12.57	37
OZ211710.1	12	12.45	36.50
OZ211711.1	13	12.20	37
OZ211712.1	14	12.14	37.50
OZ211713.1	15	12.03	37
OZ211714.1	16	11.69	37
OZ211715.1	17	11.45	37.50
OZ211716.1	18	11.03	37
OZ211717.1	19	10.66	37
OZ211718.1	20	10.46	37.50
OZ211719.1	21	10.30	37.50
OZ211720.1	22	8.88	36.50
OZ211721.1	23	8.85	37
OZ211722.1	24	8.54	37.50
OZ211723.1	25	7.84	37
OZ211724.1	26	7.54	37.50
OZ211725.1	27	7.20	38
OZ211726.1	28	6.68	39
OZ211727.1	29	6.25	37
OZ211698.1	Z	41.51	37

The mitochondrial genome was also assembled (length 16.01 kb, OZ211728.1). This sequence is included as a contig in the multifasta file of the genome submission and as a standalone record.

### Assembly quality metrics

**Figure 4.**
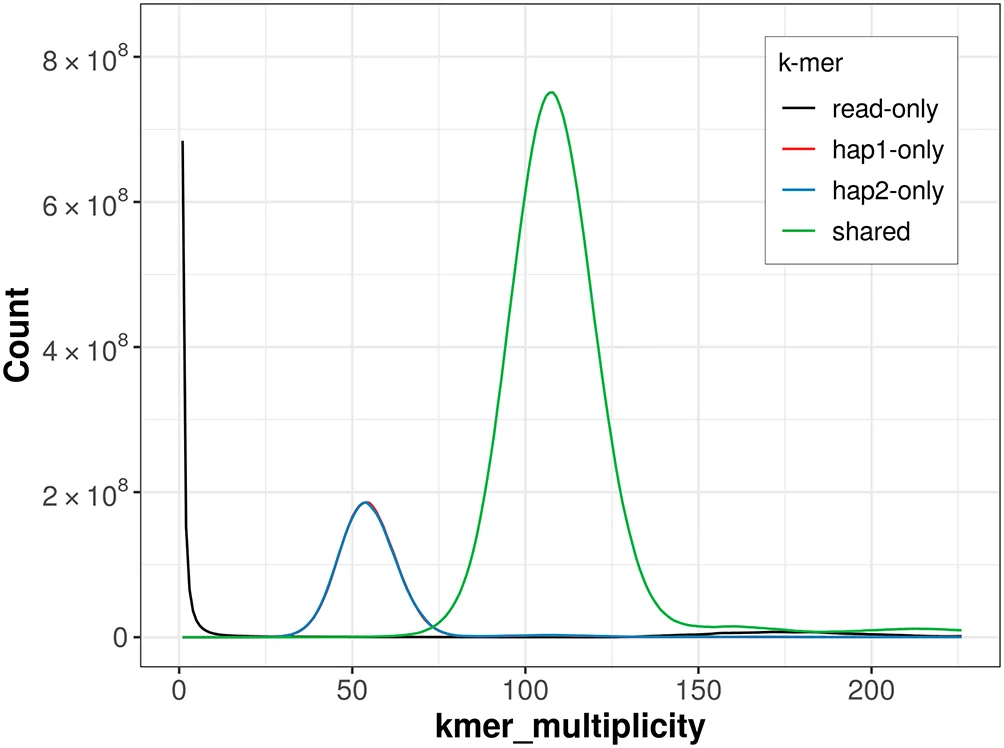
Evaluation of *k*-mer completeness using MerquryFK. This plot illustrates the recovery of *k*-mers from the original read data in the final assemblies. The horizontal axis represents *k*-mer multiplicity, and the vertical axis shows the number of *k*-mers. The black curve represents *k*-mers that appear in the reads but are not assembled. The green curve corresponds to *k*-mers shared by both haplotypes, and the red and blue curves show *k*-mers found only in one of the haplotypes.

For haplotype 1, the estimated QV is 67.4, and for haplotype 2, 67.7. When the two haplotypes are combined, the assembly achieves an estimated QV of 67.5. The *k*-mer completeness is 79.84% for haplotype 1, 79.84% for haplotype 2, and 99.09% for the combined haplotypes ( Figure 4).

**Figure 5.**
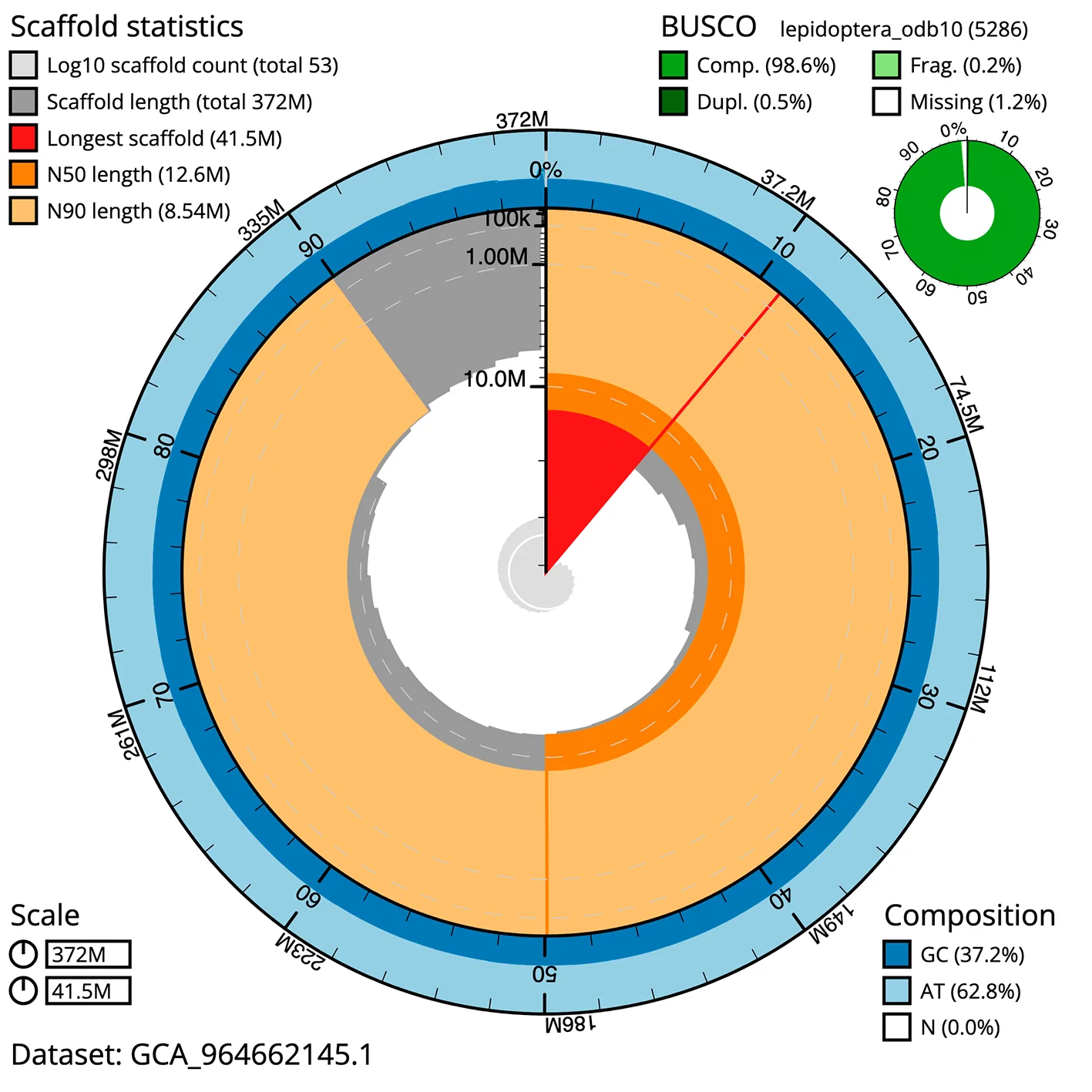
Assembly metrics for ilEanInca1.hap1.1. The BlobToolKit snail plot provides an overview of assembly metrics and BUSCO gene completeness. The circumference represents the length of the whole genome sequence, and the main plot is divided into 1 000 bins around the circumference. The outermost blue tracks display the distribution of GC, AT, and N percentages across the bins. Scaffolds are arranged clockwise from longest to shortest and are depicted in dark grey. The longest scaffold is indicated by the red arc, and the deeper orange and pale orange arcs represent the N50 and N90 lengths. A light grey spiral at the centre shows the cumulative scaffold count on a logarithmic scale. A summary of complete, fragmented, duplicated, and missing BUSCO genes in the set is presented at the top right. An interactive version of this figure can be accessed on the BlobToolKit viewer.

**Figure 6.**
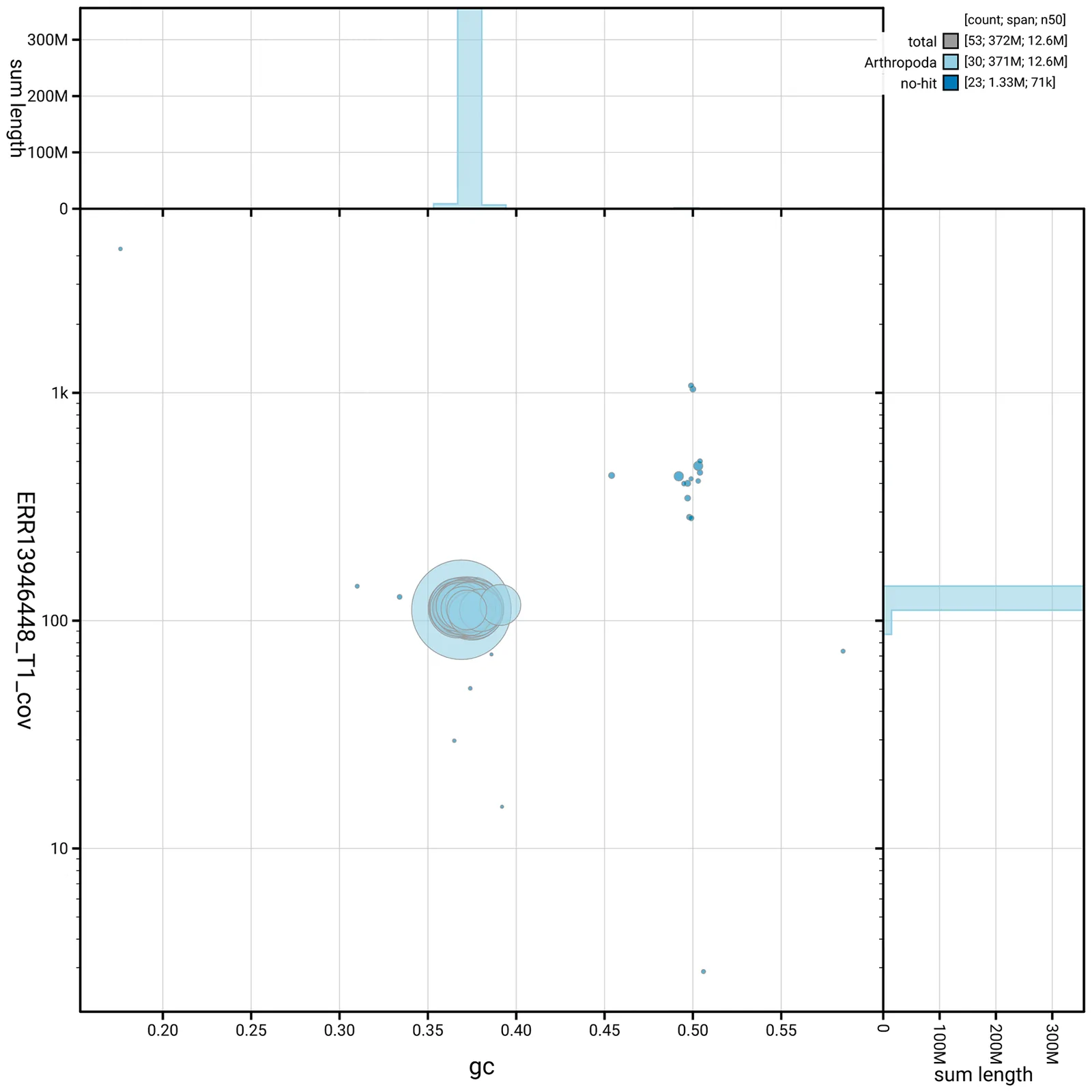
BlobToolKit blob plot for ilEanInca1.hap1.1. The plot shows base coverage (vertical axis) and GC content (horizontal axis). The circles represent scaffolds, with the size proportional to scaffold length and the colour representing phylum membership. The histograms along the axes display the total length of sequences distributed across different levels of coverage and GC content. An interactive version of this figure is available on the BlobToolKit viewer.

BUSCO analysis using the lepidoptera_odb10 reference set ( *n* = 5 286) identified 98.6% of the expected gene set (single = 98.1%, duplicated = 0.5%) in haplotype 1. For haplotype 2, BUSCO v.5.5.0 analysis identified 98.5% of the expected gene set (single = 98.1%, duplicated = 0.5%). The snail plot in Figure 5 summarises the scaffold length distribution and other assembly statistics for haplotype 1. The blob plot in Figure 6 shows the distribution of scaffolds by GC proportion and coverage for haplotype 1.

Table 4 lists the assembly metric benchmarks adapted from Rhie *et al.* (2021) and the Earth BioGenome Project Report on Assembly Standards January 2026. The EBP metric, calculated for the haplotype 1, is **6.C.Q67**, meeting the recommended reference standard.

**Table 4.** Earth biogenome project summary metrics for the *Eana incanana* assembly.

Measure	Value	Benchmark
EBP summary (haplotype 1)	6.C.Q67	6.C.Q40
Contig N50 length	6.34 Mb	≥ 1 Mb
Scaffold N50 length	12.57 Mb	= chromosome N50
Consensus quality (QV)	Haplotype 1: 67.4; haplotype 2: 67.7; combined: 67.5	≥ 40
*k*-mer completeness	Haplotype 1: 79.84%; Haplotype 2: 79.84%; combined: 99.09%	≥ 95%
BUSCO	C:98.6% [S:98.1%, D:0.5%], F:0.2%, M:1.2%, n:5 286	S > 90%; D < 5%
Percentage of assembly assigned to chromosomes	99.64%	≥ 90%

**Notes:** The EBP summary uses log10(Contig N50); chromosome-level (C) or log10(Scaffold N50); Q (Merqury QV). BUSCO: C = complete; S = single-copy; D = duplicated; F = fragmented; M = missing; n = orthologues

**Table 5.** Software versions and sources used for *Eana incanana.*

Software	Version	Source
BLAST	2.14.0	ftp://ftp.ncbi.nlm.nih.gov/blast/executables/blast+/
BlobToolKit	4.3.9	https://github.com/blobtoolkit/blobtoolkit
BUSCO	5.5.0	https://gitlab.com/ezlab/busco
bwa-mem2	2.2.1	https://github.com/bwa-mem2/bwa-mem2
DIAMOND	2.1.8	https://github.com/bbuchfink/diamond
fasta_windows	0.2.4	https://github.com/tolkit/fasta_windows
FastK	1.1	https://github.com/thegenemyers/FASTK
GenomeScope2.0	2.0.1	https://github.com/tbenavi1/genomescope2.0
Gfastats	1.3.6	https://github.com/vgl-hub/gfastats
Hifiasm	0.19.8-r603	https://github.com/chhylp123/hifiasm
HiGlass	1.13.4	https://github.com/higlass/higlass
MerquryFK	1.1.2	https://github.com/thegenemyers/MERQURY.FK
Minimap2	2.24-r1122	https://github.com/lh3/minimap2
MitoHiFi	3	https://github.com/marcelauliano/MitoHiFi
MultiQC	1.14; 1.17 and 1.18	https://github.com/MultiQC/MultiQC
Nextflow	23.10.0	https://github.com/nextflow-io/nextflow
PretextSnapshot	0.0.5	https://github.com/sanger-tol/PretextSnapshot
PretextView	1.0.3	https://github.com/sanger-tol/PretextView
samtools	1.19.2	https://github.com/samtools/samtools
sanger-tol/ascc	0.1.0	https://github.com/sanger-tol/ascc
sanger-tol/blobtoolkit	0.6.0	https://github.com/sanger-tol/blobtoolkit
sanger-tol/curationpretext	1.4.2	https://github.com/sanger-tol/curationpretext
Seqtk	1.3	https://github.com/lh3/seqtk
Singularity	3.9.0	https://github.com/sylabs/singularity
TreeVal	1.4.0	https://github.com/sanger-tol/treeval
YaHS	1.2a.2	https://github.com/c-zhou/yahs

## Author information

Contributors are listed at the following links:

• Members of the Natural History Museum Genome Acquisition Lab
• Members of the Darwin Tree of Life Barcoding collective
• Members of the Wellcome Sanger Institute Tree of Life Management, Samples and Laboratory team
• Members of Wellcome Sanger Institute Scientific Operations – Sequencing Operations
• Members of the Wellcome Sanger Institute Tree of Life Core Informatics team
• Members of the Tree of Life Core Informatics collective
• Members of the Darwin Tree of Life Consortium

## Wellcome sanger institute – legal and governance

The materials that have contributed to this genome note have been supplied by a Darwin Tree of Life Partner. The submission of materials by a Darwin Tree of Life Partner is subject to the **‘Darwin Tree of Life Project Sampling Code of Practice’**, which can be found in full on the Darwin Tree of Life website. By agreeing with and signing up to the Sampling Code of Practice, the Darwin Tree of Life Partner agrees they will meet the legal and ethical requirements and standards set out within this document in respect of all samples acquired for, and supplied to, the Darwin Tree of Life Project. Further, the Wellcome Sanger Institute employs a process whereby due diligence is carried out proportionate to the nature of the materials themselves, and the circumstances under which they have been/are to be collected and provided for use. The purpose of this is to address and mitigate any potential legal and/or ethical implications of receipt and use of the materials as part of the research project, and to ensure that in doing so we align with best practice wherever possible. The overarching areas of consideration are:

• Ethical review of provenance and sourcing of the material
• Legality of collection, transfer and use (national and international)

Each transfer of samples is further undertaken according to a Research Collaboration Agreement or Material Transfer Agreement entered into by the Darwin Tree of Life Partner, Genome Research Limited (operating as the Wellcome Sanger Institute), and in some circumstances, other Darwin Tree of Life collaborators.

## Data Availability

European Nucleotide Archive: Eana incanana. Accession number PRJEB82141. The genome sequence is released openly for reuse. The *Eana incanana* genome sequencing initiative is part of the Darwin Tree of Life Project (PRJEB40665), the Sanger Institute Tree of Life Programme (PRJEB43745) and Project Psyche (PRJEB71705). All raw sequence data and the assembly have been deposited in INSDC databases. The genome will be annotated using available RNA-Seq data and presented through the Ensembl pipeline at the European Bioinformatics Institute. Raw data and assembly accession identifiers are reported in Tables 1 and 2. Production code used in genome assembly at the WSI Tree of Life is available at https://github.com/sanger-tol . Table 5 lists software versions used in this study.
