# Supplementary material for: The genome sequence of the Bluebell moth, Eana incanana (Stephens, 1852) (Lepidoptera: Tortricidae)
Source: Wellcome Open Res. 2026 May 11;11:275. [Version 1] doi: 10.12688/wellcomeopenres.26549.1 (PMC13550895; doi:10.12688/wellcomeopenres.26549.1)
Supplement: Peer review history

# Reviewer report: Reviewer response for version 1

**DOI:** 10.21956/wellcomeopenres.29240.r159034

**Published:** 2026-07-10

**Authors:** Jonathan Badger

**Affiliations:** 1 Laboratory of Integrative Cancer Immunology, National Cancer Institute Center for Cancer Research, Bethesda, Maryland, USA

The authors report on their assembly of the genome of a male Bluebell moth. Of the two haplotypes obtained, one included the Z sex chromosome. The assembly statistics look fine and I have no concerns about the data or analysis (which follows the suggested analysis for Darwin Tree of Life projects).

Are sufficient details of methods and materials provided to allow replication by others?

Yes

Is the rationale for creating the dataset(s) clearly described?

Yes

Are the datasets clearly presented in a useable and accessible format?

Yes

Are the protocols appropriate and is the work technically sound?

Yes

Reviewer Expertise:

I am a computational genomicist, primarily in a biomedical context, although I have also contributed to the analysis of insect genomes.

I confirm that I have read this submission and believe that I have an appropriate level of expertise to confirm that it is of an acceptable scientific standard.

# Reviewer report: Reviewer response for version 1

**DOI:** 10.21956/wellcomeopenres.29240.r159037

**Published:** 2026-06-27

**Authors:** Gulab D Khedkar

**Affiliations:** 1 Dr Babasaheb Ambedkar Marathwada University, Aurangabad, Maharashtra, India

The manuscript is well prepared and presents a high-quality chromosome-level genome assembly of the Bluebell moth, *Eana incanana *(Stephens, 1852) *, *generated using PacBio HiFi sequencing technologies. The assembly metrics are considerably good ( *k *= 31), with high contiguity, completeness, and consensus quality, meeting the recommended standards for reference genomes. The methods are clearly described, the data are thoroughly validated, and all sequencing datasets and assembly resources are made publicly available, ensuring reproducibility and broad utility. The study provides a valuable genomic resource for future investigations into the evolution, comparative genomics, ecology, and adaptation of Tortricidae moths and will be of lasting interest to the research community. The manuscript is clearly written, logically organized, and appropriate for a genome data report. I did not identify any issues that warrant revision.

**Recommendation:** I recommend this manuscript for acceptance in its current form.

Are sufficient details of methods and materials provided to allow replication by others?

Yes

Is the rationale for creating the dataset(s) clearly described?

Yes

Are the datasets clearly presented in a useable and accessible format?

Yes

Are the protocols appropriate and is the work technically sound?

Yes

Reviewer Expertise:

Population genetics, genomics, DNA Barcoding

I confirm that I have read this submission and believe that I have an appropriate level of expertise to confirm that it is of an acceptable scientific standard.

# Reviewer report: Reviewer response for version 1

**DOI:** 10.21956/wellcomeopenres.29240.r155905

**Published:** 2026-05-27

**Authors:** Haoran Lu

**Affiliations:** 1 Zunyi Medical University, Zunyi, China

This is a high-quality genome data report representing the standard output level of the Darwin Tree of Life project. The authors have successfully assembled a chromosome-level reference genome of the Bluebell moth, *Eana incanana*, using PacBio HiFi long-read sequencing combined with Hi-C chromosome conformation capture technology. The data quality is exceptionally high, fully meeting and even exceeding the recommended standards of the Earth BioGenome Project (EBP). The indexing of this genome provides a valuable resource for comparative genomics of Lepidoptera insects, chromosome evolution, and studies on plant-insect chemical defense interactions. I recommend acceptance after minor revision.

 1.The current description of the core characteristic of *E. incanana*—"selective metabolism of glycosidase inhibitors"—is overly brief. It is recommended to supplement: previous biochemical research progress on this characteristic and existing limitations; specific scientific questions that can be addressed after obtaining a high-quality genome (e.g., identifying key gene families involved in DMDP metabolism, analyzing the evolutionary history of these genes). It is suggested to add a section introducing the current status of genomics research in the family Tortricidae, explaining the representativeness of the *E. incanana* genome and the gaps it fills in this family.

 2.As a data note, although in-depth functional analysis is not required, it is recommended to add a brief comparative genomics section comparing basic characteristics of *E. incanana* such as genome size, chromosome number, GC content, and repeat sequence content with published closely related species (e.g., other species in the tribe Cnephasiini or model species in Tortricidae). In particular, regarding chromosome number, *E. incanana* has 29 autosomes plus the Z sex chromosome, which differs from the typical karyotype of Lepidoptera insects (n=31). It is suggested to discuss the possible evolutionary significance of this difference.

 3. Extending this comparison to include other agriculturally important insect pests from different families and orders would provide broader insights into the genomic basis of insect adaptation to diverse ecological niches. For example, comparing with the codling moth *Cydia pomonella* (Wan et al., 2019), a congener of *E. incanana* and a major invasive pest of pome fruits, would be particularly valuable for understanding the genomic evolution within the Tortricidae family. Additionally, including high-quality chromosome-level genomes of other key pests such as the cowpea beetle *Callosobruchus maculatus* (Lu et al., 2024), the sweetpotato whitefly *Bemisia tabaci* (Xie et al., 2021), the Colorado potato beetle *Leptinotarsa decemlineata* (Yan et al., 2023), and the diamondback moth *Plutella xylostella* (Boyes et al., 2023) would allow for a more comprehensive comparative analysis of pest-specific genomic features, such as gene family expansions related to host adaptation, detoxification, and chemical defense tolerance. In particular, regarding chromosome number, *E. incanana* has 29 autosomes plus the Z sex chromosome, which differs from the typical karyotype of Lepidoptera insects (n=31). It is suggested to discuss the possible evolutionary significance of this difference in the context of karyotype evolution across different pest species.

 4.The article only briefly mentions in the results section that "a haplotypic inversion was observed on Chromosome Z in the region ~12.94–14.25 Mb" without any analysis or discussion. It is recommended to: (1) Describe the size of this inversion region and the number of genes it contains; (2) Discuss the potential role of chromosomal inversions in species differentiation and adaptive evolution of Lepidoptera; (3) Explain the implications of this finding for future research.

 5.The current description of the mitochondrial genome is limited to the single sentence "with a length of 16.01 kilobases", which provides too little information. It is suggested to supplement: the gene composition and arrangement order of the mitochondrial genome; comparative analysis with mitochondrial genomes of other closely related species; phylogenetic analysis based on the mitochondrial genome to further verify the taxonomic status of *E. incanana*.

 6.Currently, only a ventral photograph of the specimen is provided in Fig. 1. It is recommended to add a dorsal photograph. Species identification of Tortricidae insects is mainly based on the dorsal markings of the forewings, and a dorsal photograph is crucial for subsequent researchers to verify the specimen identity.

 7.The article mentions that the DNA barcode of this sample belongs to BIN cluster BOLD:AAD7476. It is suggested to supplement the number of sequences included in this BIN, their geographical distribution range, and detailed genetic distance information from closely related species.

 8.MIn the "Assembly curation" section, it is recommended to briefly explain the judgment basis for performing manual breaks, joins, and removal of duplicates.

Are sufficient details of methods and materials provided to allow replication by others?

Yes

Is the rationale for creating the dataset(s) clearly described?

Yes

Are the datasets clearly presented in a useable and accessible format?

Yes

Are the protocols appropriate and is the work technically sound?

Yes

Reviewer Expertise:

Genomics, Entomology, Chemical ecology, Innate immunity

I confirm that I have read this submission and believe that I have an appropriate level of expertise to confirm that it is of an acceptable scientific standard, however I have significant reservations, as outlined above.

## References

[1] : A chromosome-level genome assembly of Cydia pomonella provides insights into chemical ecology and insecticide resistance. *Nature Communications* .2019;10(1) : 10.1038/s41467-019-12175-9 10.1038/s41467-019-12175-9 PMC674899331530873

[2] : High-quality reference genome of cowpea beetle Callosobruchus maculatus. *Scientific Data* .2024;11(1) : 10.1038/s41597-024-03638-w 10.1038/s41597-024-03638-w 39025902 PMC11258224

[3] : Genome sequencing of the sweetpotato whiteflyBemisia tabaci MED/Q. *GigaScience* .2017;6(5) : 10.1093/gigascience/gix018 10.1093/gigascience/gix018 PMC546703528327996

[4] : Chromosome-level genome assembly of the Colorado potato beetle, Leptinotarsa decemlineata. *Scientific Data* .2023;10(1) : 10.1038/s41597-023-01950-5 10.1038/s41597-023-01950-5 36653371 PMC9849343

[5] : The genome sequence of the Diamondback Moth, Plutella xylostella (Linnaeus, 1758). *Wellcome Open Research* .2023;8: 10.12688/wellcomeopenres.20006.1 10.12688/wellcomeopenres.20006.1 PMC1110971038779055
